# Supplementary material for: Utilization of community engagement in social innovation health projects in low-and-middle income countries: A global sequential mixed methods analysis
Source: PLOS Glob Public Health. 2026 Mar 18;6(3):e0006055. doi: 10.1371/journal.pgph.0006055 (PMC12998873; doi:10.1371/journal.pgph.0006055)
Supplement: S1 Table — (DOCX) [file pgph.0006055.s001.docx]

**S1Table: Socio-demographic characteristics of the key informant participants involved in identified social innovations (N=27)**

| Characteristic | Frequency |
| --- | --- |
| Sex of participant |  |
| Male | 16 |
| Female | 11 |
| Country |  |
| Africa | 18 |
| Asia | 4 |
| Latin America and the Caribbean | 5 |
| Health focus of the innovation |  |
| PHC | 14 |
| MCH | 5 |
| HIV/STDs | 4 |
| Neglected Tropical Diseases | 3 |
| Malaria | 3 |
| Infectious disease | 2 |
| General | 1 |
| Community Health | 1 |
| Health System Focus |  |
| Service delivery | 11 |
| Health care financing | 3 |
| Community service delivery | 5 |
| Health workforce | 4 |
| Information systems | 3 |
| Others | 11 |

**Health system focus** - Leadership/governance, Medical resources, Referral system, Community Empowerment, Community mobilization, Health Insurance, Human resources for health, Information, Telemedicine, Technology, Medical products and technologies
