# Supplementary material for: Utilization of community engagement in social innovation health projects in low-and-middle income countries: A global sequential mixed methods analysis
Source: PLOS Glob Public Health. 2026 Mar 18;6(3):e0006055. doi: 10.1371/journal.pgph.0006055 (PMC12998873; doi:10.1371/journal.pgph.0006055)
Supplement: S1 File — (DOCX) [file pgph.0006055.s002.docx]

## S1 File: Description of Social Innovation in Health Case Studies

| **Name of the Social Innovation in Health** | **Description** |
| --- | --- |
| LifeNet International | **Implementer**: LifeNet International  **Operations**: Burundi, Democratic Republic of Congo, Uganda  **Organizational** **structure**: NGO  **Challenge**: Burundi suffers from poor quality of basic health care service provision.  **Solution**: LifeNet partners with church-based health centres to provide them with the necessary tools to expand the scope of their services, while holding them accountable to quality standards. Each LifeNet partner health centre retains full ownership of their facility and works with the LifeNet team to implement programme components specific to their needs. The LifeNet franchise bundle includes medical and management staff training, quality evaluations, supply of essential pharmaceuticals and growth financing through an affordable loan scheme. |
| Schistosomiasis Control  Initiative | **Implementer**: Schistosomiasis Control Initiative at Imperial  College London  **Operations**: Ethiopia, Burundi, Cote D’Ivoire, Democratic Republic of Congo, , Liberia, Madagascar, Malawi, Mauritania, Mozambique, Niger, Rwanda, Senegal, Sudan, Tanzania, Uganda, Yemen, Zambia, Zanzibar  **Organizational** structure: University  **Challenge**: Even though 200 million people in Africa are infected with schistosomiasis, awareness of its consequences is low and access to medication is limited.  **Solution**: The SCI works to decrease the incidence of schistosomiasis in Africa by mapping the distribution of the disease across the continent in order to inform and advocate appropriate policies in partnership with the ministries of health. The SCI advocates for free treatment, often provided through drug donations by pharmaceutical companies and raises funds  to facilitate free delivery of the drugs to patients. The initiative includes training of healthcare workers and teachers to deliver the drugs and health education to communities. |
| Safe Water and Aids Project | **Implementer**: Safe Water and AIDS Project (SWAP)  **Operations**: Kenya  **Organization** **structure**: NGO  **Challenge**: Kenya has a high burden of disease where poor health is exacerbated by poor water quality and inadequate sanitation.  **Solution**: SWAP enables community health promoters (CHPs) to conduct door-to-door sales and health education to improve health. They also increase access to health and hygiene products for vulnerable communities. SWAP provides training and support for CHPs to promote healthy practices and generate income. The product mix includes water treatment, ceramic filters, hand wash soaps, detergents, diapers, sanitary pads, de-worming tablets, fortified flour, micro-nutrient powder, clean cook stoves, mosquito nets and condoms. |
| Riders for Health | **Implementer**: Riders for Health  **Operations**: Lesotho, Kenya, Malawi, Nigeria, The Gambia, Zambia, Zimbabwe  **Organizational** **structure**: NGO (Social enterprise)  **Challenge**: One of the most overlooked barriers to health care delivery is a lack of reliable, well-maintained transport.  **Solution**: Riders for Health works with partner organizations to manage fleets of motorcycles and four-wheeled vehicles that are used by health workers to reach the most remote communities with health care. These workers are trained and mobilized to cover large distances, taking health care directly to these communities rather than waiting for them to come to the health facility. Training equips them to ride, maintain and repair their own motorcycles. Riders for Health also utilizes its transport network to facilitate supply chain distribution. |
| Last Mile Health | **Implementer**: Last Mile Health  **Operations**: Liberia  **Organizational** **structure**: NGO  **Challenge**: In Liberia, people often lack access to basic health care because they live too far from a health facility, up to 30 kilometres away across difficult terrain.  **Solution**: Last Mile Health recruits, trains, equips, manages and pays professionalised community health workers (CHWs) to provide primary health care in the last mile. CHWs are members of the communities they serve, selected by their own leaders to receive training because of their talent, commitment and ability. Last Mile Health works closely with community members, local government officials, national policymakers, private sector and global partners to develop and scale the model sustainably. |
| Learner Treatment Kit | **Implementer**: Save the Children Malawi, London School of Hygiene & Tropical Medicine, Malaria Alert Centre (University of Malawi), Malawi Ministries of Health and Education  **Operations**: Malawi  **Organizational** **structure**: Partnership  **Challenge**: Malaria is a major contributor to school absenteeism, with schoolchildren being most commonly infected but least likely to have access to treatment.  **Solution**: Teachers are trained to use rapid diagnostic tests to diagnose and treat schoolchildren with malaria and other common illnesses through Learner Treatment Kit (LTK) right in the classroom. LTK is a box containing rapid diagnostic tests for malaria, antimalarial medicines and other basic health supplies. This brings education and health care together, simultaneously keeping children healthy while learning. |
| Kaundu Community-Based Health  Insurance | **Implementer**: Kaundu Health Centre (Christian Health Association of Malawi) and Community **Operations**: Malawi  **Organizational structure**: NGO and Community  **Challenge**: Rural communities with pay-for-use health facilities often have limited access to quality health services due to the cost barrier.  **Solution**: A community-initiated and owned health insurance scheme reduces out-of-pocket expenditure associated with seeking health services at Kaundu Health Centre. The scheme provides financial protection against the unforeseen cost of illness. This is a bottom-up communitydriven approach with defined community roles that support the delivery and accountability of the solution, expanding access to health services and enhancing local governance. |
| GP Down-Referral Model | **Implementer**: BroadReach Healthcare, North-West Province Department of Health and KOSHMED **Operations**: South Africa  **Organizational** **structure**: Company and Provincial Government  **Challenge**: About 6.4 million South Africans are living with HIV, of which 2 million are eligible for antiretroviral therapy but not yet on treatment.  **Solution**: The General Practitioner (GP) referral model is a public-private partnership to increase access to antiretroviral treatment services. The model leverages the excess capacity of private GP’s to provide services to public patients who are stable on their antiretroviral treatment. It aids to reduce the burden on over-crowded government hospitals, freeing up capacity of public hospitals to initiate treatment in new patients or manage complicated cases. |
| Pharmacist Assistant  Programme | **Implementer**: Keth’Impilo  **Operations**: South Africa  **Organizational** **structure**: NGO  **Challenge**: South Africa currently has 1 pharmacist for every 3 849 people, with less than 30% practicing in the overburdened public sector.  **Solution**: The General Practitioner (GP) referral model is a public-private partnership to increase access to antiretroviral treatment services. The model leverages the excess capacity of private GP’s to provide services to public patients who are stable on their antiretroviral treatment. It aids to reduce the burden on over-crowded government hospitals, freeing up capacity of public hospitals to initiate treatment in new patients or manage complicated cases. |
| Action For Women and  Awakening In Rural Environment | **Implementer**: Action For Women and Awakening In Rural Environment (AWARE)  **Operations**: Uganda  **Organizational structure**: NGO  **Challenge**: Women in Karamoja suffer from high levels of gender-based violence; poor access to education; unemployment; poor financial services; limited access to health care; and a lower voice in decision-making compared to men.  **Solution**: AWARE aims to advance the health, social, cultural and economic wellbeing of women and girls in Karamoja through utilizing a holistic approach to empower women and advocate for their rights in the community. Women are equipped with agricultural and business skills and are sensitized on their rights. AWARE has established a multipurpose women’s centre, which has a maternity waiting house where expectant mothers can receive health care services and life skill training. |
| Bwindi Mothers’ Waiting Hostel | **Implementer**: Bwindi Community Hospital  **Operations**: Uganda  **Organizational structure**: NGO  **Challenge**: Although well-established medical care exists to prevent maternal deaths, most women in remote and hard-to-reach areas cannot access this care.  **Solution**: Bwindi Mothers’ Waiting Hostel identifies high risk mothers living in hard-to-reach areas through the hospital’s community nurse team. These women are then encouraged to come and stay in the hostel for up to a month before delivery, depending on the severity of their risk. Daily monitoring of the mothers is done by midwives. Supervised deliveries, antenatal services, counselling, emergency obstetric care, and education services are provided to the mothers. |
| Drug Shops Integrated Care | **Implementer**: Makerere University  **Operations**: Uganda  **Organizational** **structure**: University  **Challenge**: Many households receive care from local drug shops, which vary in quality and ability to offer health services.  **Solution**: The Drug Shop Integrated Care programme aims to improve the quality of services in private drug shops by adopting the WHO/UNICEF strategy for integrated Community Case Management in standardizing care. Through the programme, shop attendants are trained to recognize malaria, pneumonia and diarrhoea. They can then deliver the appropriate diagnostics |
| Imaging the World Africa | **Implementer**: Imaging the World Africa  **Operations**: Uganda  **Organizational structure**: Social enterprise  **Challenge**: Most rural pregnant mothers cannot access obstetric imaging service in Uganda, due to the insufficient number of radiologists and sonographers within the health system. Patients often travel long distances to access such services at public hospitals; or incur high costs at urban private clinics.  **Solution**: Imaging the World, Africa trains registered nurses and midwives working in rural health facilities to be competent in performing antenatal ultrasound scans. Through new technology, the scans can be uploaded electronically and sent via a cellular data network to radiologists abroad to aid with real time interpretation. |
| Kyaninga Child Development  Centre | **Implementer**: Kyaninga Child Development Centre (KCDC)  **Operations**: Uganda  **Organizational structure**: NGO  **Challenge**: In Uganda, more than 12% of children are living with disabilities (CWDs). The children have disproportionately unequal opportunities for basic needs and stigma Is severe, which discourages them from seeking health care.  **Solution**: KCDC provides a holistic approach to care for children living with disabilities, and their families, in rural western Uganda. KCDC provides rehabilitative services including physiotheraphy, occupational therapy and speech therapy at a minimal or no cost to children. 70% of the services are delivered in the community - homes, schools or local health centres. Through training programmes and peersupport structures, parents are equipped with the skills and confidence to care for their child at home. Innovative funding mechanisms are adopted to contribute towards sustainability. |
| Mamatoto Approach | **Implementer**: Healthy Child Uganda  **Operations**: Uganda  **Organizational structure**: University programme  **Challenge**: Maternal and child mortality remain very high in Uganda, with 368 maternal deaths per 100,000 live births and 64 child deaths per 1000 live births occurring annually (UBoS 2016), most of which are preventable.  **Solution**: MamaToto is a district-led programme that operationalizes the Village Health Team (VHT) strategy and includes health system strengthening to promote quality maternal, new-born and child health (MNCH) practices. The district leaders develop, implement and monitor their own MNCH priorities in partnership with a network of community health volunteers who conduct home visits, assess and refer patients, provide health education, and mobilize communities to participate in health activities. |
| The Medical Concierge Group | **Implementer**: The Medical Concierge Group Limited  **Operations**: Uganda  **Organization structure**: Company  **Challenge**: In Uganda, the lack of qualified medical personnel (especially in rural areas), along with rising costs of out-of-pocket expenses, present significant barriers for many to accessing health care. **Solution**: The Medical Concierge Call Centre provides telemedicine consultations and social media health messaging that is free, accessible anytime and operates in multiple languages through multiple platforms such as voice, SMS, Facebook, Twitter, WhatsApp, Skype and email. Through the call centre, people in urban and rural areas have access to health care information and services provided by licensed doctors and pharmacists. |
| Living Goods | **Implementer**: Living Goods  **Operations**: Kenya, Uganda  **Organizational structure**: NGO  **Challenge**: Community health workers (CHWs) play a critical role in low-resource settings, but volunteer CHW programmes often suffer from low productivity and high attrition rates.  **Solution**: Living Goods provides ongoing training, financial support, access to quality treatments and products, performance incentives and mobile technology to villagebased health entrepreneurs. They go door-to-door offering relevant health information and selling health-orientated products. An initial loan from Living Goods allows them to earn a modest income, whilst delivering basic health care services. |
| Everyday Family Health Plan | **Implementer**: Philippine Health Insurance Corporation, Palawan Local Health Insurance Office (LHIO) **Operations**: Philippine  **Organizational structure**: Government Agency  **Challenge**: Despite a national health insurance programme in the Philippines (PhilHealth), there is low collection rate of health insurance premiums, and therefore low coverage, among members of the informal sector in the Philippines, translating to poor access to health services. This is precipitated by the informal sector’s low and irregular income, and lack of technical capability to manage a savings mobilization program.  **Solution**: “P6.60” is a savings mobilization scheme that aims to increase the insured health coverage of informal sector workers under PhilHealth. In a month, TODA members are required to save a minimum amount of P6.60 per day, the equivalent of USD 0.13. Once their savings reach the required quarterly PhilHealth premium, the group treasurer remits the full amount to PhilHealth. Members also benefit from medical check-ups and financial management seminars. |
| National Telehealth System (NTS) | **Implementer**: National Telehealth Center, National Institutes of Health, University of the Philippines Manila  **Operations**: Philippine  **Organizational structure**: University  **Challenge**: There are approximately 4,720 communities in the Philippines that are considered geographically isolated and disadvantaged areas (GIDAs). Health professionals that work in GIDAs are not only faced with limited financial and logistical resources, but also a shortage of critical health human resources to ensure the delivery of quality health care services.  **Solution**: NTS is a telemedicine platform and training programme that leverages ubiquitous technology such as short messaging services (SMS) or emails to improve access to specialist care among rural health workers. The innovation is in the secure referral network that the project created, linking rural health physicians to clinical specialists in selected regional hospitals nationwide. |
| Seal of Health Governance  (SOHG) | **Implementer**: Municipality of Del Carmen  **Operations**: Philippine  **Organizational structure**: Local Government Unit  **Challenge**: Key health challenges faced by the island municipality of Surigao Del Norte include low facility-based delivery, high number of maternal deaths, malnutrition, poor nutrition, and high incidence of infectious and vector-borne diseases such as tuberculosis, schistosomiasis and dengue.  **Solution**: The SOHG is a monitoring and evaluation program that ensures quality implementation and sustainability of health programmes of the municipality by empowering leadership and governance capacities of community leaders. The program provides annual financial and in-kind incentives to the best performing villages of the Municipality and awards the Seal of Health Governance to the winning barangays (districts) |
| Noora Health | Implementer: Noora Health Operations: India Organisational structure: NGO CHALLENGE: In resource-constrained settings, relapse and complications following discharge from hospital is common. SOLUTION: Noora Health trains patients and their families with high-impact health skills to improve outcomes and save lives. They turn hospital hallways and waiting rooms into classrooms. Through engaging, skill-based learning, Noora Health ensures that patients’ families are equipped to deal with the consequences of severe diagnoses, surgery or childbirth, replacing anxiety with competency and easing the transition from hospital to home. They rely on nurse educators, ensuring continuity through a ‘train-the-trainer’ approach |
| Social Entrepreneurship for Sexual Health | **Implementer**: Social Entrepreneurship for Sexual Health (SESH) Global  **Operations**: People’s Republic of China  **Organizational structure**: University  **Challenge**: HIV testing rates are low in China and sexual health messaging tends to be old fashioned and unengaging.  **Solution**: SESH is a multi-sectoral research collaboration that utilizes creative contributory contests to crowdsource sexual health messaging that is directly informed by the lives and experiences of the target population. This ‘bottomup’ approach taps into the wisdom of crowds to generate appropriate and engaging materials. It allows for greater inclusion of perspectives from diverse community members and possesses higher potential for innovation – compared to conventional expert-led approaches. |
| Comprehensive Health Approach for Chagas Disease in Comapa | **Implementer**: Universidad del Valle de Guatemala (UVG)  **Operations**: Guatemala  **Organizational structure**: University Programme  **Challenge**: Comapa is a region in southwestern Guatemala that has approximately 28,000 of habitants and showed the highest rates of infestation and seropositivity of Chagas disease in children throughout the country, in addition to conditions of poverty and low quality of life. **Solution**: The programme offers a contexually acceptable and comprehensive approach to the awareness, prevention, diagnosis and treatment of Chagas disease. Through active engagement and participation, communities are facilitated through a process to identify their key needs and design solutions. This process takes place in partnership with state health insitututions and NGO’s in Comapa (Jutiapa, Guatemala). |
| Eco-Health Approach to Fight Chagas Disease | **Implementer**: Laboratory of Applied Entomology and Parasitology (LENAP) at Universidad de San Carlos  **Operations**: Guatemala  **Organizational structure**: University programme  **Challenge**: Chagas disease is associated with conditions of extreme poverty because the insect transmitting the parasite infects households in poor rural communities where cheap, readily accessible materials such as adobe, wood and palm leaves are used for construction, and animals are reared inside houses.  **Solution**: The Eco-health approach pursues sustainable prevention of Chagas’ disease through: 1) the design of a strategy to fill the cracks in the floors and walls using a scientifically tested combination of locally available materials; and 2) increasing awareness of these risks through training of leaders and members of the community to repair and improve their own homes and to adopt the healthier practice of raising animals outside of the household. |
| Mothers of the River Programme | **Implementer**: Universidad Peruana Cayetano Heredia  **Operations**: Peru  **Organizational structure**: University  **Challenge**: The department of Loreto in the Peruvian Amazon has one of the highest maternal and infant mortality rates in Peru. Up to 80% of women give birth at home and infection is a leading cause of neonatal death. Health facilities are difficult to access and most are understaffed, poorly equipped, and often lack basic infrastructure.  **Solution**: Community health workers (CHW) and traditional birth attendants are trained to promote essential newborn care practices during home deliveries, in case facility-based delivery is not feasible. CHW conduct regular prenatal and postnatal home visits to mothers. During these visits, they give out printed materials and use tablet computers with a health application to help monitor the health status of mothers and newborns and provide educational content related to maternal and newborn health. The information collected is share with the corresponding health agencies by the CHW. |
| SMS-Hub Leprosy Case Management System | **Implementer**: The Leprosy Mission Mozambique, Mozambique Health Department.  **Operations**: Mozambique  **Organisation structure**: NGO  **Challenge**: In Mozambique, hand-written records (often disorganised or incomplete) are used to monitor the management of people with leprosy, making the flow of information difficult and unreliable.  **Solution**: The SMS-Hub is an SMS-based notification system that improves the management, monitoring and evaluation of leprosy. It allows district, provincial and national leprosy supervisors to track cases using their own phone. They can gather data to improve case management, planning and resource allocation, and monitor stock levels at district facilities to prevent drug stock-outs. The system can be accessed from any place with mobile reception and can send detailed information to mobile or email. |
| Centre for the Development of Scientific Research | **Implementer**: Centre for the Development of Scientific Research (CEDIC)  **Operations**: Paraguay.  **Organizational** **structure**: NGO  **Challenge**: Indigenous communities of the Paraguayan Chaco have a high prevalence of Chagas disease and poor living conditions.  **Solution**: CEDIC uses the living labs methodology to engage community members, public administration, universities, research centres and industry partners to codesign social innovations that tackle Chagas disease health challenges. Projects include building vector-resistant housing, distributing games to teach children about Chagas prevention and partnering with a local enterprise to deliver safe drinking water and drought-resistant agricultural practices. |
