# Supplementary material for: Utilization of community engagement in social innovation health projects in low-and-middle income countries: A global sequential mixed methods analysis
Source: PLOS Glob Public Health. 2026 Mar 18;6(3):e0006055. doi: 10.1371/journal.pgph.0006055 (PMC12998873; doi:10.1371/journal.pgph.0006055)
Supplement: Attachment — Submitted filename: Response Letter to the Review Comments.docx [file pgph.0006055.s004.docx]

**Makerere University School of Public Health,**

**New Mulago Hill Road, Mulago Kampala.**

**Tel: +256 774 734803 / +256 704 734803**

**Email:** [eahumuza@musph.ac.ug](mailto:eahumuza@musph.ac.ug)

January 13, 2026

**The Academic Editor and Reviewers,**

**PLOS Global Public Health.**

Dear Sirs/Madams,

**REF: Responses to the review comments for the Manuscript titled, ‘Community Engagement in Social Innovation Health Projects: A Global Sequential Mixed Methods Analysis’**

First and foremost, we greatly and humbly thank you all for taking off time to carefully read our manuscript and giving us very insightful review comments. We greatly believe that addressing these comments has been very key in improving our manuscript. Thank you very much once again.

We hereby humbly submit the responses to the different review comments raised, as shown below;

A. **REVIEWER ONE**
1. **General**

The relationship between community engagement (CE) and social innovation (SI) is not quite clear. The writing should provide clarity on whether CE facilitates social innovation or SE improves CE. The clarity here would determine backgrounds where problems/gaps are stated, the methods, findings and discussions.

- Thank you very much for this very insightful guidance. The introduction has been completely revised to address this key comment, as shown from Lines 53 to 111. Thanks.

2. **Introduction**
a) The argument "This approach [SI] prioritizes bottom-up development, empowering communities to actively shape health programs and services, thereby enhancing sustainability and accountability1. Social innovations differ from technical innovations by focusing on societal challenges through participatory solutions rather than market-oriented products or technologies". This creates a confusion where CE inherently has similar characteristics. So, which is which? what is the relationship between CE and SI? Which one contributes and benefits or what is the dual connection between the two?

- This is well noted with much appreciation. The introduction has been completely revised, and all confusing statements have been removed, as shown from Lines 53 to 111. A clear relationship between SI and CE has been added. Thank you very much.

b) Further in the background section there is a statement that reads as "Robust community engagement is a foundation of social innovation. Community engagement can be used to tailor social innovations, accelerate implementation research, facilitate pragmatic clinical trials, inform local health policy, and help sustain social innovation over time". This fuels the confusion over the connection between CE and SI. The ladders of CE is widely documented to be inform, consult, involve, collaborate and empower (Harvard Catalyst. Community engagement: Innovation and improvement in public health via community engagement and research (nd).

Am not sure if the ladder of CE for SI is different from these and why? Note - available evidence have established CE aims to empower the community to own and lead the change process. Similarly, the connection between community engagement and social innovation health projects needs to be well articulated. What is the role of CE and SI in health projects and what makes such projects innovative?

Is it CE or SI components of the project and consequent outcome or both?

That said, the introduction section needs to clarify existing experiences and gaps that may have called to document this which is grossly missing and in fact contributed to crude objective.

- Thank you for this comment. The introduction has been completely revised, and all confusing statements have been removed, as shown from Lines 53 to 111. Thank you very much.

3. **Objective**
a) "The purpose of this study was to examine and understand community engagement in social innovation in health projects in LMICs". This is too general "examine and understand" what of CE and SI and in which health projects?

- The objective of the study has been revised and narrowed down, as shown in Lines 110 to 112. Thank you for this comment.

4. **Methods**
a) Sequential mixed method approach was used which is great. Nonetheless, except a mention of "The initial qualitative data informed the selection of items in the subsequent quantitative survey", it is not clear what elements of data were sought with the initial qualitative method to inform the survey.

- Thanks for this comment. This has been addressed, as shown in lines 116 to 124. Thanks.

b) Neither was it clear on what were the measurements in the survey except a very general statement that reads, "...it allows for a more comprehensive understanding of community engagement in social innovations in health..... focused on social innovators, government officials, researchers, and other stakeholders who have experience with social innovation in health... The survey included domains on socio-demographic characteristics, community engagement breadth and depth, facilitators and barriers of community engagement, and related topics". These needs to be fleshed in terms of a) why quali was done before and to generate what type of variables for survey and what the survey intended to measure b) What pre selection were used to select cases for the interviews from the global compendium of 40 social innovation case studies needs to be clearer c) specific details to such statements as: community engagement breadth and depth" including how these were measured.

- This has been addressed, as shown in lines 116 to 153. Thank you very much

c) Although the design gives an impression to inform selection of items for the survey, there are findings from qualitative which offers an impression that the qualitative part of this study goes beyond informing the selection of items as stated in the method section. This needs to be corrected.

- This is a good comment, and the description of the study design has been revised to address this comment, as shown in lines 116 to 124. Thank you very much

5. **Results**

a) While I believe result section could be improved with clarity on some the comments above, I would love to know more about what Table 3 in the result section intends to impart? Why multiple responses? what is the difference between stage at which community engagement was utilized and community engagement utilized in SI? What does assess community engagement in social innovations mean? Check number of participants 369 against 274???

- Thanks for this comment. Table 3 aims to show the stage of the social innovation at which community engagement was utilized, and the community engagement activities utilized. This has been clarified in the manuscript, as shown in lines 202 to 203.

‘What is the difference between stage at which community engagement was utilized and community engagement utilized in SI?’ This has also been addressed.

For assessing community engagement, it looked at whether social innovations conducted an assessment for community engagement in the social innovations, to understand its impact. This has also been revised for more clarity, as shown in lines 203 to 204.

‘369’ was a clerical error, and has been corrected. For this table, it was 269 participants. Thank you very much.

b) The qualitative findings need to align with the theme of the finding which should be aligned with the overarching questions (hopefully this could be clarified by further specifying the objectives.

- Thank you very much for this comment. However, we wrote our qualitative results together with the different sections of the quantitative results because the qualitative results compliment the quantitative results, and in some instances have deeper understanding. Hence, we think separating them and aligning them to their different themes might make them to look the connection with the rest of the looks (loss of chronological flow). Thank you very much.

6. **Discussion and conclusion**

a) It is important to identify some topical finding from this study and interpret each with available evidence or experiences. I am not sure if "Our findings show that half of the social innovation health projects had a very high-level engagement, categorized as either “shared leadership” or “collaborate”" While the qualitative findings and quotes do not suggest this. You may need to check your data.

- This is a very insightful comment, and it has been addressed, as shown in lines 356 to 359. Thank you very much.

b) The statement in the conclusion section "...this study demonstrates moderate to high community engagement across various stages of social innovation in health" doesn't seem have foundation. Neither measurements were clearer in the method section nor, the findings demonstrated this.

- Thank you very much for this comment. We have revised our conclusion to address this comment, as shown in lines 425 to 430. Thanks.

B. **REVIEWER TWO**

a) The manuscript is well organized, presented, and written in Standard English. The results of the research are well discussed in the light of the available related literature and the conclusions are supported by data drawn from mixed methods analysis.

- This is well noted. Thank you very much

b) However, the title as well as the purpose of the manuscript needs to be specific in terms of geographical scope (such as LMICs as indicated in the manuscript).

- This has been addressed, as shown in lines 1 to 3. Thank you very much.

c) Moreover, the authors need to clearly show the originality of the results of their research based on exhaustive and critical review of available related literature

- Thank you very much for this very insightful comment. A thorough literature review has been done, as shown in the revised introduction section of the manuscript from lines 52 to 112. Thank you very much.

Once again, thank you very much for the comments.

**Yours faithfully,**

**Emmanuel Ahumuza.**
